# Incarcerated workers: overlooked as essential workers

**DOI:** 10.1186/s12889-022-12886-7

**Published:** 2022-03-15

**Authors:** Marjorie Naila Segule, Katherine LeMasters, Meghan Peterson, Michael Forrest Behne, Lauren Brinkley-Rubinstein

**Affiliations:** 1 Environmental Health Department, Harvard T.H. Chan School of Public Health, Harvard University, Cambridge, MA USA; 2 Department of Epidemiology, Gillings School of Global Public Health, University of North Carolina at Chapel Hill, Chapel Hill, NC USA; 3 School of Social Medicine, Center for Health Equity Research, University of North Carolina at Chapel Hill, Chapel Hill, NC USA

**Keywords:** Prison labor, COVID-19, Occupational health, Incarcerated workers

## Abstract

**Objective:**

To use the example of COVID-19 vaccine prioritization for incarcerated workers to call attention to the need to prioritize incarcerated workers’ health.

**Methods:**

From November to December 2020, we searched publicly available information (e.g. Department Of Corrections websites and press releases) for 53 US prison systems, including all states, Immigration and Customs Enforcement, the Federal Bureau of Prisons, and Puerto Rico. Coders reviewed if states had prison labor policies, if states had COVID-19 specific prison labor policies, the location of work, industries both pre- and during the COVID-19 pandemic, the scope of work, and hourly wage. Findings were compared to the Centers for Disease Control and Prevention’s occupational vaccine prioritization recommendations.

**Results:**

Every facility has incarcerated individuals working in some capacity with some resuming prison labor operations to pre-pandemic levels. All but one prison system has off-site work locations for their incarcerated population and many incarcerated workers have resumed their off-site work release assignments. Additionally, every state has incarcerated workers whose job assignments are considered frontline essential workers (e.g. firefighters). In at least five states, incarcerated workers are participating in frontline health roles that put them at higher risk of acquiring COVID-19. Yet, no state followed the Centers for Disease Control and Prevention recommended vaccination plan for its incarcerated population given their incarcerated workers’ essential worker status.

**Conclusion:**

The Centers for Disease Control and Prevention recommended that incarcerated people be prioritized for vaccination primarily due to the risk present in congregate style prison and jail facilities. Furthermore, our review found that many incarcerated people perform labor that should be considered “essential”, which provides another reason why they should have been among the first in line for COVID-19 vaccine allocation. These findings also highlight the need for incarcerated workers’ health to be prioritized beyond COVID-19.

**Supplementary Information:**

The online version contains supplementary material available at 10.1186/s12889-022-12886-7.

## Background

The Centers for Disease Control and Prevention’s (CDC’s) Advisory Committee on Immunization Practices (ACIP) listed people who are incarcerated at increased risk of COVID-19 acquisition and transmission and has thus identified them as a critical population deserving of COVID-19 vaccine prioritization [1]. Yet, only 34 states and territories included this population in their Phase 1 vaccine distribution [2].

One of the four risk categories to determine vaccine prioritization is the risk of negative societal impact [1]. This criterion assesses the impact that a population becoming infected with COVID-19 would have on the functioning of society. The ACIP has not scored incarcerated populations in this area, even though many incarcerated individuals provide labor in industries that have been deemed essential to maintaining societal function [3]. This work sheds light on prison labor in the Federal Bureau of Prisons, U.S. Immigration and Customs Enforcement (ICE), Puerto Rico and the 50 state prison systems and incarcerated workers’ prioritization for the COVID-19 vaccine. It also speaks to the need to prioritize incarcerated workers’ health beyond the context of COVID-19.

Incarcerated workers are an overlooked population in workplace protections even though half of those incarcerated in federal and state prisons work full-time [4]. For example, most incarcerated workers are unable to file Occupational Safety and Health Administration (OSHA) complaints. Between February and October 2020, there was a 15% increase in OSHA workplace complaints, a significant proportion coming from essential workers and focusing on inadequate COVID-19 workplace conditions [5]. Yet, the specific needs of incarcerated workers went unheard even though many were involved in the front lines of the COVID-19 response. Incarcerated workers who are able to file OSHA complaints, are not allowed employee representatives and OSHA inspections are significantly controlled by prison administration such as bans on cameras, incarcerated workers only being interviewed with institutional approval, and prison staff are informed before an OSHA inspection, which is illegal in non-incarcerated settings [6].

The 13th Amendment outlawed slavery except as a punishment for crime, which enables incarcerated individuals to be forced to work as part of their sentence without proper workplace protections [7, 8]. Given their work requirements, it is critical for incarcerated workers to be provided with safe workplace protections during the pandemic and to have been prioritized for the COVID-19 vaccine.

## Methods

Using prison systems’ department websites, press releases, social media pages, and news articles, we identified whether each of the 53 prison systems has a prison labor policy, a COVID-19 specific prison labor policy, the location of work, industries both pre- and during the COVID-19 pandemic, the scope of work, and hourly wage. This data was collected between November and December 2020.

Two coders independently searched for information and a third coder reconciled differences. The finalized industries were compared to the CDC’s recommendation for occupational vaccine prioritization to identify which states have listed incarcerated individuals as essential workers and the relevant corresponding vaccination phase (1A, 1B, 1C) [9]. Phase 1A has been used for those who are working in frontline COVID-19 healthcare jobs that are at increased risk for acquiring COVID-19 such as working in mortuary services, washing hospital laundry, and cleaning medical units. Phase 1B is for frontline essential workers (e.g., firefighting, agriculture, manufacturing) and Phase 1C is for other essential workers (e.g., food services, construction, transportation). The COVID-19 Prison Labor Policies have been summarized below in Table 1.

**Table 1:** Prison System COVID-19 Prison Labor Policies

Types of COVID-19 Prison Labor Policies	Number of Systems
Developed a COVID-19 Specific Prison Labor Policy	32
Suspended all off-site incarcerated work operations during the pandemic	12
Included Social Distancing in their COVID-19 Specific Prison Labor Policy	3
Assigned Incarcerated Workers into Housing Units by Work Assignments	3
Pivoted job assignments to different industries (e.g. manufacturing personal protective equipment)	38
Prioritized Incarcerated Workers for the COVID-19 Vaccination	0

## Results

The full data from this research is available in the attached supplemental material (see Additional File 1) [10]. While the CDC recommended vaccination plan for prioritizing essential industries includes industries that hire incarcerated workers, no states publicly prioritized incarcerated individuals by their work assignments [9].

For Phase 1A, eight states have had incarcerated workers employed as frontline health workers pre- and during the pandemic. Five states of these eight states (Michigan, Missouri, New York, Oregon, Texas) have confirmed that incarcerated workers may be exposed to COVID-19 through work in mortuary services and cleaning hospital laundry.

Every state has incarcerated workers whose job assignments are considered frontline essential workers under Phase 1B. The most common industries for incarcerated workers were manufacturing, agriculture, firefighting, and meat processing. Twelve states have incarcerated workers who participate in other essential services such as food services and construction.

Every state has a prison labor policy, however, 18 states (Alabama, Arkansas, Colorado, Illinois, Kentucky, Massachusetts, Michigan, Mississippi, Missouri, Montana, Nebraska, Nevada, New Hampshire, New York, North Dakota, Ohio, Oklahoma, Rhode Island) did not have a COVID-19 specific prison labor policy. Many states pivoted to manufacturing personal protective equipment and increased sanitation jobs due to the COVID-19 pandemic. Only three systems (Maryland, ICE, Washington) made specific mention of social distancing in their COVID-19 specific prison labor policies. Three systems (Arizona, Louisiana, Puerto Rico) moved incarcerated workers into the same housing unit based on work assignments. However, it is unclear to what extent these policies are enforced.

Incarcerated workers have continued working both on-site and off-site at the federal and state levels. During our search in November to December 2020, twelve systems had suspended all off-site work details in their most recent policy update, while the other states had continued or resumed.

There is significant variation in hours across systems and wages. Most facilities have a maximum of forty hours a week in line with full-time work in the general public. However, some allow up to 60 h a week or 12 h a day. Many states required deductions from wages for court debts, taxes, mandatory savings, or charge for custody and care (also called room and board). Only those working in mortuary services have been given pay increases for COVID-19 facing work. In New York, workers transitioned from an average hourly wage of 65¢ to $6 for digging mass graves. In Texas, workers who were previously unpaid received $2 per hour to work in the mobile morgues.

## Conclusions

Prior literature has advocated for incarcerated individuals being prioritized for COVID-19 vaccinations given the high-risk environments of congregate, over-crowded settings [11]. There has been slow vaccine roll-out for incarcerated individuals as a whole. In May 2021, most states’ incarcerated population had a lower first dose vaccination rate compared to the general public; with the exception of Alaska, Arizona, California, Colorado, Delaware, Massachusetts, Tennessee, and Virginia [12].

We argue that in addition to prioritizing COVID-19 vaccination for all incarcerated persons, incarcerated workers must be prioritized in alignment with their job. While recent efforts, such as in New York, have sped up the vaccination of incarcerated individuals, the prioritization of incarcerated workers specifically has not occurred [13].

The main limitation of this study was relying on publicly available information as we are unable to verify the implementation of COVID-19 labor policies in carceral settings and the collected data is not specific enough for differentiating between occupations and industries. We found examples of changes in prison labor protocols without an update on COVID-19 prison labor policies. Further research should involve interviewing incarcerated workers on their experiences working before and during the pandemic. Additionally, COVID-19 vaccine prioritization and roll-out are ever-changing. It is possible that our information is out-of-date at the time of publication. Nonetheless, it is important to highlight that as vaccinations were approved and prioritization was assigned at the end of 2020, incarcerated workers were absent from state plans.

## Public health implications

This research further illustrates the already high need for incarcerated individuals to be prioritized for the COVID-19 vaccine as many incarcerated individuals work at jobs that are high risk for transmission and exposure to COVID-19. This research also highlights that incarcerated workers’ voices are not typically included in workplace safety or occupational health surveillance tools like OSHA complaints. Future research is needed for the assessment of prison labor policies and health outcomes during and beyond the COVID-19 pandemic at the prison system level.

Furthermore, to better prepare for future health crises and potential vaccination campaigns, incarcerated workers must be fully recognized as essential workers and be prioritized as such.

## Supplementary Information

**Additional file 1.**

## Data Availability

All data coded or analyzed during this study are included in this published article and in the attached supplementary materials.

## Abbreviations

CDC: Centers for disease control and prevention
ACIP: Advisory committee on immunization practices
ICE: Immigration and customs enforcement
OSHA: Occupational safety and health administration
